# Coprological and molecular prevalence of *Cryptosporidium* and *Giardia* in cattle and irrigation water from Beni-Suef Governorate, Egypt

**DOI:** 10.1038/s41598-025-10552-7

**Published:** 2025-07-24

**Authors:** Fatma El-zahraa Ramadan Saleh, Hend H. A. M. Abdullah, Dina Aboelsoued

**Affiliations:** 1 Parasitology Laboratory, Water Pollution Research Department, Environmental and Climate Change Institute, National Research Centre, Giza, 12622 Egypt; 2 Department of Parasitology and Animal Diseases, Veterinary Research Institute, National Research Centre, Dokki, Giza, 12622 Egypt

**Keywords:** *Cryptosporidium hominis*, *Cryptosporidium ubiquitum*, *Cryptosporidium ryanae*, *Cryptosporidium bovis*, *Giardia* assemblage A, Risk factors, Molecular biology, Diseases, Infectious diseases

## Abstract

*Cryptosporidium* and *Giardia* are globally significant protozoan parasites responsible for severe foodborne and waterborne outbreaks, posing substantial zoonotic and environmental risks. This study aimed to comprehensively assess the prevalence of cryptosporidiosis, giardiasis, and co-infections in Beni-Suef Governorate, Egypt, using an integrated diagnostic approach combining microscopy and molecular techniques. Additionally, it was sought to identify associated risk factors in cattle fecal samples. Microscopical examination of 970 cattle fecal samples revealed an overall infection rate of 67.42% (654/970), with *Cryptosporidium* detected in 42.68% (414/970), *Giardia* in 11.96% (116/970), and co-infections in 12.78% (124/970) of cases. In irrigation water, *Cryptosporidium* oocysts and *Giardia* cysts were detected in 2/24 (8.33%) and 1/24 (4.16%) of samples, respectively. Molecular and phylogenetic analyses identified *Cryptosporidium hominis* in cattle and, for the first time in Egypt, *Cryptosporidium ubiquitum* and *Cryptosporidium ryanae* in irrigation water, while also proving the presence of *Cryptosporidium bovis* and *Giardia* assemblage A in cattle. Risk factors, including sex, age, season, and fecal consistency, significantly influenced infection rates, with higher prevalence in females, calves under two months, spring season, and diarrheic feces. These findings underscore the urgent need for One Health-based control strategies, integrating targeted interventions to mitigate the burden of *Cryptosporidium* and *Giardia* infections and environmental contamination.

**Supplementary Information:**

The online version contains supplementary material available at 10.1038/s41598-025-10552-7.

## Introduction

*Cryptosporidium* and *Giardia* are globally significant protozoan parasites frequently associated with severe foodborne and waterborne outbreaks^1,2^. These pathogens contribute substantially to morbidity and mortality worldwide, particularly in low-resource settings where they disproportionately impact vulnerable populations, including children, immunocompromised individuals, and newborn animals^3–6^. Both parasites exhibit a wide range of vertebrate hosts, infecting humans, livestock, wildlife, and birds, causing self-limited diarrhea alongside other clinical manifestations^7–11^. Their zoonotic potential and diverse transmission pathways, including zoonotic, foodborne, and waterborne routes, underscore their relevance as a critical One Health concern, highlighting the interconnectedness of human, animal, and environmental health^12–14^.

*Cryptosporidium* oocysts and *Giardia* cysts are highly resilient in the environment, persisting in diverse matrices such as soil, water, and food. Their transmission is facilitated through contaminated tap water, bottled water, surface water, ground water, and irrigation systems, posing significant public health and environmental challenges^15–18^. Farm animals, particularly cattle, play a pivotal role in the epidemiology of these protozoa, as young calves serve as major reservoirs, shedding millions of infectious oocysts and cysts into the environment, contaminating water sources, and amplifying the zoonotic transmission risks^8,19–21^. The remarkably low infective dose of *Giardia*, fewer than 10 cysts, further exacerbates the ease of transmission, complicating public health and environmental management efforts^20–22^. The infective dose of *Cryptosporidium parvum* ranges from 5.8 to 16.6 oocysts^23^ yet a single infected host can shed over 3 × 10^10^ oocysts into the environment^24^ creating a significant potential reservoir for environmental contamination^25^. To date, more than 40 *Cryptosporidium* spp. have been identified in mammals, reptiles, birds, fish, and amphibians^26^. Of the approximately 20 species known to infect humans, *C. parvum* and *Cryptosporidium hominis* are the most prevalent, accounting for over 90% of human cases globally^3^. In cattle, the predominant species include *C. parvum*, *Cryptosporidium bovis*, *Cryptosporidium andersoni*, and *Cryptosporidium ryanae*^26^. Among these, *C. parvum* is particularly notable for its broad host range and significant zoonotic potential. *Giardia intestinalis* (synonyms: *Giardia lamblia* and *Giardia duodenalis*) is a species complex comprising eight genetically distinct assemblages (A–H). Assemblages A and B are of particular concern due to their low host specificity, allowing them to infect both humans and a wide variety of animal species^22,27,28^. Co-infections involving *Cryptosporidium* and *Giardia* are increasingly documented in both humans and animals, particularly in regions with inadequate sanitation or environmental contamination^20,29,30^. Emerging evidence suggests that co-infections may exacerbate clinical severity, leading to prolonged diarrhea, persistent inflammation, and malnutrition, especially in newborn animals^31,32^. The concurrent presence of both pathogens within a host may compromise immune responses, potentially facilitating the colonization or persistence of one pathogen by the other, thereby complicating disease management and treatment outcomes^30^. Furthermore, their simultaneous detection in environmental matrices, such as irrigation water and soil, underscores the heightened risk of widespread contamination and transmission^10,15^.

Cryptosporidiosis is endemic in cattle worldwide, with reported prevalence rates ranging from 11.7 to 78%, particularly affecting pre-weaned calves^33–36^. In humans, the global prevalence of cryptosporidiosis has been estimated at 14.1% in high-income countries and up to 31.5% in low-income countries^37,38^. Human infections with *Giardia* spp. are similarly widespread, ranging from 2 to 5% in developed nations to 20–30% in developing regions^39–42^. In cattle, the pooled prevalence of giardiasis has been estimated at 24% based on microscopic examination^43^. Both *Cryptosporidium* and *Giardia* are well-recognized as major pathogens responsible for numerous foodborne and waterborne outbreaks^1,44–46^. Globally, the proportion of waterborne outbreaks attributed to *Cryptosporidium* increased markedly to 77.4% between 2017 and 2022, while those caused by *Giardia* declined significantly to 17.1% during the same period^47^.

Cryptosporidiosis and giardiasis pose significant One Health challenges due to their intricate interplay across human, animal, and environmental systems^13,14^. Accurate diagnosis requires a combination of microscopical and molecular techniques to identify species and genotypes, which are critical for understanding host specificity, pathogenicity, and zoonotic potential^30,48,49^. Elucidating the prevalence, risk factors, and potential synergistic effects of co-infection is essential for designing effective surveillance, prevention, and control strategies within the One Health framework^7,32,50^.

This study aimed to comprehensively assess the prevalence of cryptosporidiosis, giardiasis, and their co-infections in Beni-Suef Governorate, Egypt, using microscopical examination followed by genetic identification of detected species. Additionally, it seeks to identify associated risk factors in cattle fecal samples, emphasizing their role in the epidemiology of these protozoan infections.

## Materials and methods

### Study area

**Fig. 1:**
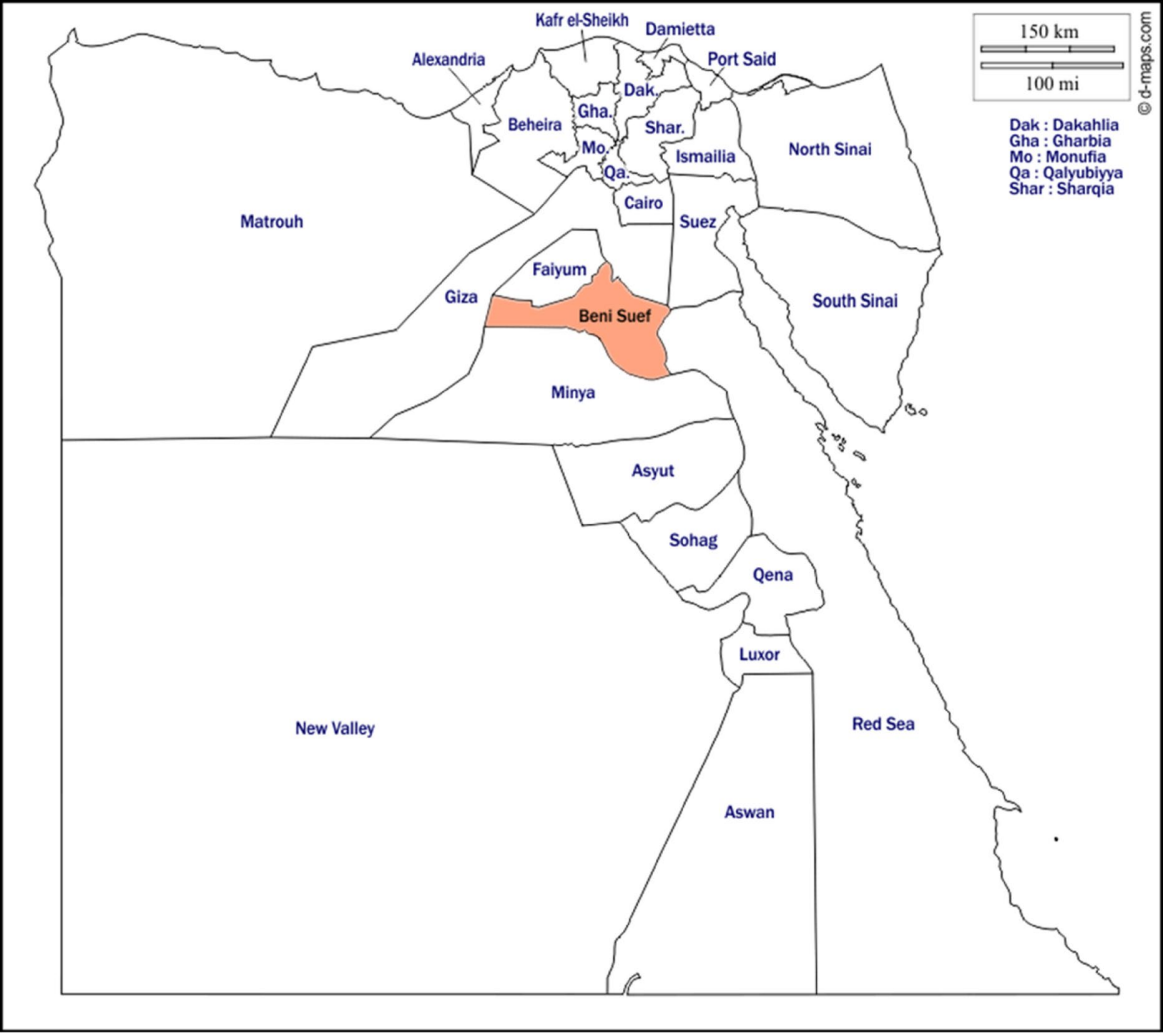
A map of Egypt showing the provinces, with Beni-Suef province highlighted in orange to indicate where the samples were collected.

The study was conducted in Beni-Suef province (29.0667° N, 31.0833° E) in northern Upper Egypt (Fig. 1), an agricultural hub where cattle rearing plays a key economic role. Cattle are raised for milk and meat, providing vital income and resources for local households. The region’s semi-arid climate, with hot summers and mild winters, shapes cattle farming practices, with farmers using irrigated pastures and crop residues for feed. The nearby Nile River ensures reliable water access, supporting cattle health and productivity.

### Sampling

A cross-sectional study was conducted in 2023 and 2024, involving 970 cattle. Fecal samples were randomly collected from cattle owned by smallholders, private farms, and at the Beni-Suef slaughterhouse. Various risk factors were recorded for each animal, including sex (female and male), age categories (< 2 months, 2–4 months, 4–6 months, and > 6 months), seasons (autumn, spring, summer, and winter), and observed fecal consistency. Rectal samples were collected from each animal and placed into clean, labeled plastic containers. The samples were then transported in an icebox to the Parasitology and Immunology Laboratory, Department of Parasitology and Animal Diseases, NRC, Egypt, on the collection day^49^. In addition, 24 irrigation water samples (20 L each) were collected from the same locations using sterile polypropylene containers. These water samples were transported to the Environmental Parasitology Laboratory, Water Pollution Research Department, NRC, Giza, Egypt, on the same day^51^.

### Parasitological examination

#### Fecal examination

##### Macroscopical examination

Fecal samples were examined macroscopically to assess consistency, color abnormalities, and the presence of blood, mucus, or other unusual components, following the methodology described by Zajac et al.^52^.

##### Microscopical examination

The fecal samples were filtered through two layers of gauze to remove large particles. Approximately 2 mg of feces was then mixed with a drop of normal saline (0.85% NaCl) and a drop of Lugol’s iodine solution, and the mixture was spread on a clean glass slide. Each specimen was examined under a light microscope (LEICA Imaging Systems Ltd., England) at 100× and 400× magnification for the morphological identification of *Giardia* cysts and trophozoites^42,49^. *Cryptosporidium* spp. were identified using the Modified Ziehl Neelsen (MZN) staining technique, as described by Henriksen and Pohlenz^53^. MZN-stained slides were examined at 400× and 1000× magnification^54^. The severity of infection was assessed by counting *Cryptosporidium* oocysts per field at 1000× magnification, following the criteria outlined by Anderson and Bulgin^55^: mild (1–5 oocysts/field), moderate (6–20 oocysts/field), and severe (more than 20 oocysts/field). Samples were stored at 4 °C in an equal volume of 2.5% potassium dichromate solution (Sigma-Aldrich, Canada) until molecular identification^56^.

#### Water examination

Each water sample was filtered using a stainless-steel pressure filter holder (Sartorius, Germany) fitted with a nitrocellulose membrane 142 mm diameter, 0.45 μm pore^57^. The membrane filters were washed three times with sterile saline, and the washing solution was centrifuged at 2000 rpm for 5 min^58^. The supernatants were discarded, and the resulting sediments were separately collected in sterile Eppendorf tubes. Parasitological examination was performed as previously described, and the samples were subsequently stored at −20 °C for molecular identification.

### Molecular screening

#### DNA extraction

DNA was extracted from heavily infected fecal and water samples that tested positive during microscopic examinations; 200 µL of each fecal sample and 500 µL of each water sample, containing concentrated oocysts, using the QIAamp^®^ DNA Stool Mini Kit (Qiagen GmbH, Hilden, Germany), following the manufacturer’s protocol. Before extraction, the samples underwent five freeze-thaw cycles, alternating between liquid nitrogen and a 95 °C water bath. The DNA concentration of each sample was measured using a Q9000 microvolume spectrophotometer (Quawell, USA). The extracted DNA was stored at − 20 °C until further analysis for pathogen screening.

#### Screening of pathogens DNA by standard PCR

All extracted DNA samples were screened using PCR with universal primers targeting the *Cryptosporidium* spp. *18S* rRNA^59^ and *Giardia* spp. *β-giardin* (*bg*) gene^60^. The PCR assays were conducted using Emerald Amp GT mastermix™ (Takara) in a BIO-RAD Thermal Cycler (BIO-RAD, Singapore). Amplification conditions for both *Cryptosporidium* and *Giardia* followed the protocols outlined by Yusof et al.^59^, Cacciò et al.^60^ (Table 1). Each PCR run included positive controls (genomic *Cryptosporidium* and *Giardia* DNA) and negative controls (molecular-grade water). Amplification products were verified by electrophoresis on a 1% agarose gel stained with Red Safe and visualized using a UV transilluminator. A 100 bp DNA ladder (Fermentas, Thermo Fisher Scientific) was used to determine the size of the PCR products.

**Table 1:** Oligonucleotide sequences of primers used for PCR and sequencing.

Pathogens	Gene name	Primer sequences	Annealing temperature	Amplicon size	References
*Cryptosporidium*	18S rRNA	CAA TTG GAG GGC AAG TCT GGT GCC AGC CCT TCC TAT GTC TGG ACC TGG TGA GT	68 °C	655 bp	^59^
*Giardia*	β-giardin	AAG CCC GAC GAC CTC ACC CGC AGT GC GAG GCC GCC CTG GAT CTT CGA GAC GAC	50 °C	753 bp	^60^

**Table 2:** Epidemiological risk factors associated with the prevalence of cryptosporidiosis and giardiasis.

Risk factors		Examined animals	Cryptosporidium mono-infections (%)	Giardia mono-infections (%)	Co-infection (%)	Overall infected animals (%)	χ^2^	*P* value
Sex	Female	511	231 (45.21%)	51 (9.98%)	66 (12.92%)	348 (68.19%)	8.45	0.0380^*^
	Male	459	183 (39.87%)	65 (14.16%)	58 (12.64%)	306 (66.66%)		
	*χ* ^***2***^_**(1)**_		5.57	1.72	0.52			
	*P* value		0.0180^*^	0.1900	0.4710			
Age	< 2 m	518	247 (47.68%)	52 (10.04%)	67 (12.93%)	366 (70.66%)	20.34	0.0160^*^
	2 to 4 m	177	68 (38.42%)	26 (14.69%)	23 (12.99%)	117 (66.10%)		
	4 to 6 m	52	17 (32.69%)	8 (15.38%)	8 (15.38%)	33 (63.46%)		
	> 6 m	223	82 (36.77%)	30 (13.45%)	26 (11.66%)	138 (61.88%)		
	*χ* ^***2***^_**(3)**_		258.12	24.83	62.23			
	*P* value		< 0.0001^*^	< 0.001^*^	< 0.0001^*^			
Season	Autumn	312	160 (51.28%)	17 (5.45%)	13 (4.17%)	190 (60.90%)	100.99	< 0.0001^*^
	Spring	220	83 (37.73%)	42 (19.09%)	56 (25.45%)	181 (82.27%)		
	Summer	221	84 (38.01%)	22 (9.95%)	27 (12.22%)	133 (60.18%)		
	Winter	217	87 (40.09%)	35 (16.13%)	28 (12.90%)	150 (69.12%)		
	*χ* ^***2***^_**(3)**_		41.21	13.72	31.42			
	*P* value		< 0.0001^*^	0.0033^*^	< 0.0001^*^			
Fecal consistency	Diarrheic	544	255 (46.88%)	60 (11.03%)	82 (15.07%)	397 (72.98%)	20.34	< 0.0001^*^
	Formed	426	159 (37.32%)	56 (13.15%)	42 (9.86%)	257 (60.33%)		
	*χ* ^***2***^_**(1)**_		22.12	0.14	12.90			
	*P* value		< 0.0001^*^	0.7110	< 0.001^*^			
Total	970		414 (42.68%)	116 (11.96%)	124 (12.78%)	654 (67.42%)		

*Indicate the presence of a statistically significant association.

#### Sequencing and phylogenetic analyses

PCR products were purified using the QIAquick PCR Purification Kit (Qiagen, Germany) following the manufacturer’s instructions. Sequencing of the purified products was carried out on an ABI 3130 automated sequencer (Applied Biosystems, USA) using the Big Dye Terminator v3.1 Cycle Sequencing Kit (Applied Biosystems). The resulting sequences were assembled and refined using the Chromas Pro program (ChromasPro 1.7, Technelysium Pty Ltd., Tewantin, Australia). After submission to GenBank, the corrected sequences for *Cryptosporidium* spp., and *Giardia* spp. were compared to existing sequences in the GenBank database using NCBI BLASTn (http://blasdt.ncbi.nlm.nih.gov/Blast.cgi). The consensus sequences were aligned with reference sequences from GenBank using CLUSTAL W v1.83^61^. Phylogenetic trees were constructed using the Maximum Likelihood method in MEGA X, based on the Tamura-Nei model, with 1,000 bootstrap replicates to ensure statistical reliability^62,63^.

### Data analysis

The impact of various risk factors, including sex, age, season, and fecal consistency, on the prevalence of *Cryptosporidium*, *Giardia*, and co-infections were assessed using the chi-square (*χ*^2^) test in SAS software, Version 9.4 (SAS Institute Inc., Cary, NC, USA). Statistical significance was determined at a threshold of *P* < 0.05.

## Results

### Prevalence of cryptosporidiosis, giardiasis, and their Co-infection

Parasitological examination revealed that out of 970 cattle examined, 654 animals (67.42%) tested positive for one or more parasitic infections. *Cryptosporidium* mono-infections had the highest prevalence, affecting 414/970 cattle (42.68%), followed by co-infections of *Cryptosporidium* with *Giardia* sp. in 124/970 cases (12.78%) and *Giardia* mono-infections in 116/970 cases (11.96%; Table 2). In irrigation water samples, *Cryptosporidium* sp. was more prevalent, detected in 2 out of 24 samples (8.33%), whereas *Giardia* sp. was identified in 1 out of 24 samples (4.16%).

### Epidemiological risk factors associated with cryptosporidiosis, giardiasis, and their Co-infection

Epidemiological analysis demonstrated that the prevalence of cryptosporidiosis was significantly associated with sex, age, seasonal variation, and fecal consistency. The highest occurrence was observed among females (45.21%; *P* = 0.0180), calves less than 2 months of age (47.68%; *P* < 0.0001), during autumn (51.28%; *P* < 0.0001), and in diarrheic cases (46.88%; *P* < 0.0001). In contrast, the prevalence of giardiasis was significantly influenced by age and seasonal variation, with the highest rates recorded in calves aged between 4 and 6 months (15.38%; *P* < 0.001) and during spring (19.09%; *P* = 0.0033). However, no statistically significant differences were found in giardiasis prevalence concerning sex or fecal consistency (Table 2). For cases of co-infection involving both pathogens, age, seasonal variation, and fecal consistency also emerged as significant determinants. The highest co-infection rates were detected in calves aged between 4 and 6 months (15.38%; *P* < 0.0001), during spring (25.45%; *P* < 0.0001), and among diarrheic cases (15.07%; *P* < 0.001). Similar to giardiasis, co-infection prevalence was not significantly associated with sex (Table 2).

Overall, the infection rates for both pathogens combined were significantly linked to all examined risk factors. Specifically, the highest prevalence was noted among females (68.19%; *P* = 0.0380), calves less than 2 months of age (70.66%; *P* = 0.0160), during spring (82.27%; *P* < 0.0001), and in diarrheic cases (72.98%; *P* < 0.0001).

### Molecular and phylogenetic analyses of cryptosporidiosis and giardiasis

**Fig. 2:**
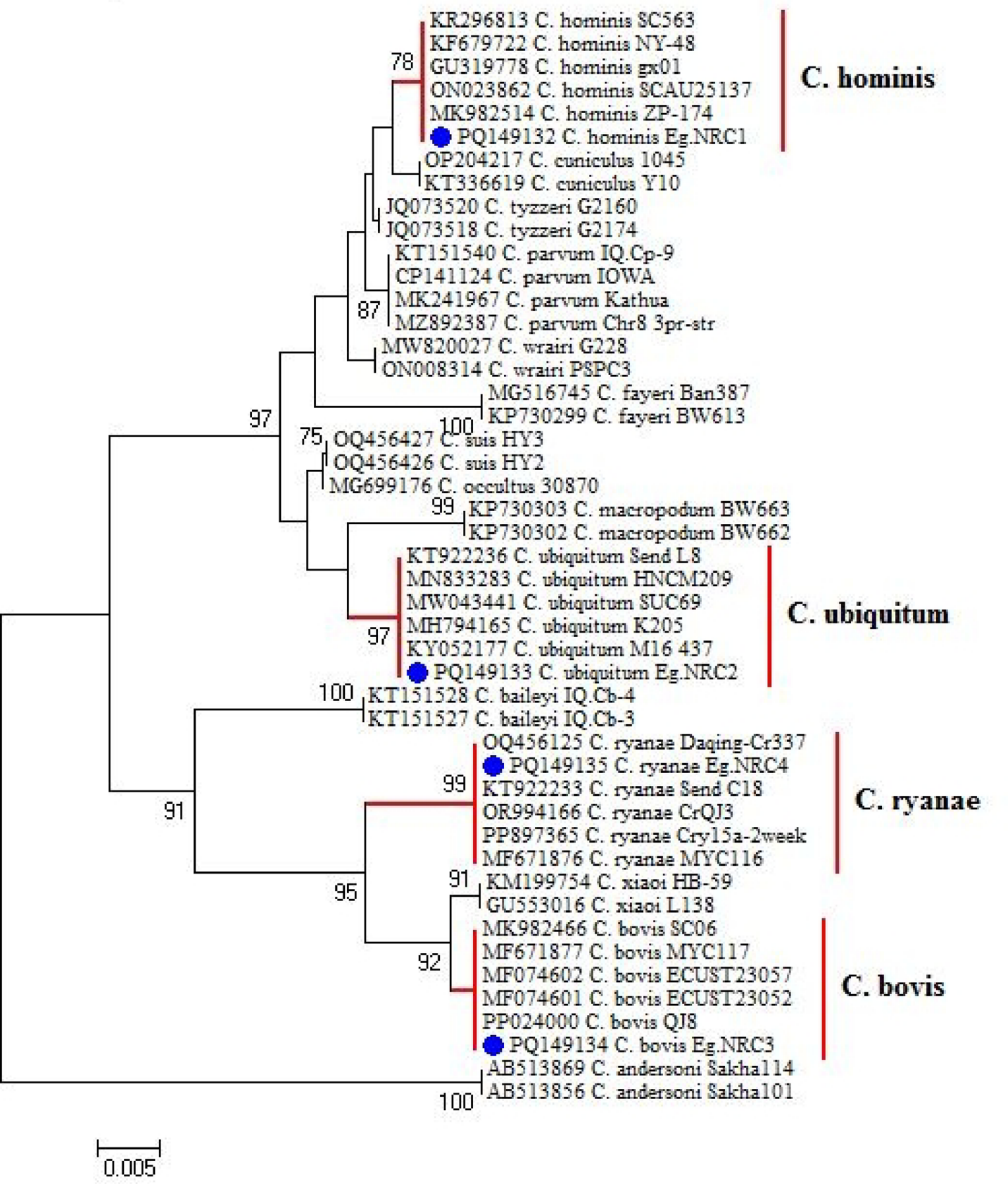
*18S* rRNA-based phylogenetic tree of *Cryptosporidium* spp. The Maximum Likelihood method was constructed based on the Tamura-Nei model with 1000 bootstrap replicates.

All microscopically positive samples were screened by species-specific primers, the *18S* rRNA gene for *Cryptosporidium* spp., and the *β-giardin* primer for *Giardia* (S1 and S2 Appendix). The *18S* rRNA gene sequencing confirmed the presence of four *Cryptosporidium* species: *Cryptosporidium hominis* and *Cryptosporidium bovis* in cattle feces, and *Cryptosporidium ubiquitum* and *Cryptosporidium ryanae* in irrigation water. BLAST analysis revealed a single genotype for each species, consistent across different seasons. *C. hominis* (GenBank: PQ149132.1) showed 100% identity (464/464 bp) with *C. hominis* detected in the feces of rhesus macaques (*Macaca mulatta*), an Old World monkey species, in Bangladesh (GenBank: MK982514). Similarly, *C. bovis* (GenBank: PQ149134.1) demonstrated 100% identity (453/453 bp) with *C. bovis* detected in the feces of dairy cattle in China (GenBank: MF074601). In irrigation water, *C. ubiquitum* (GenBank: PQ149133.1) exhibited 100% similarity (462/462 bp) to *C. ubiquitum* identified in goat feces from China (GenBank: MN833283), while *C. ryanae* (GenBank: PQ149135.1) showed 100% similarity (453/453 bp) to *C. ryanae* identified in calf feces from Ethiopia (GenBank: KT922233). Phylogenetic analysis of *Cryptosporidium* spp. indicated that our sequences clustered within the same clade as reference species (Fig. 2).

**Fig. 3:**
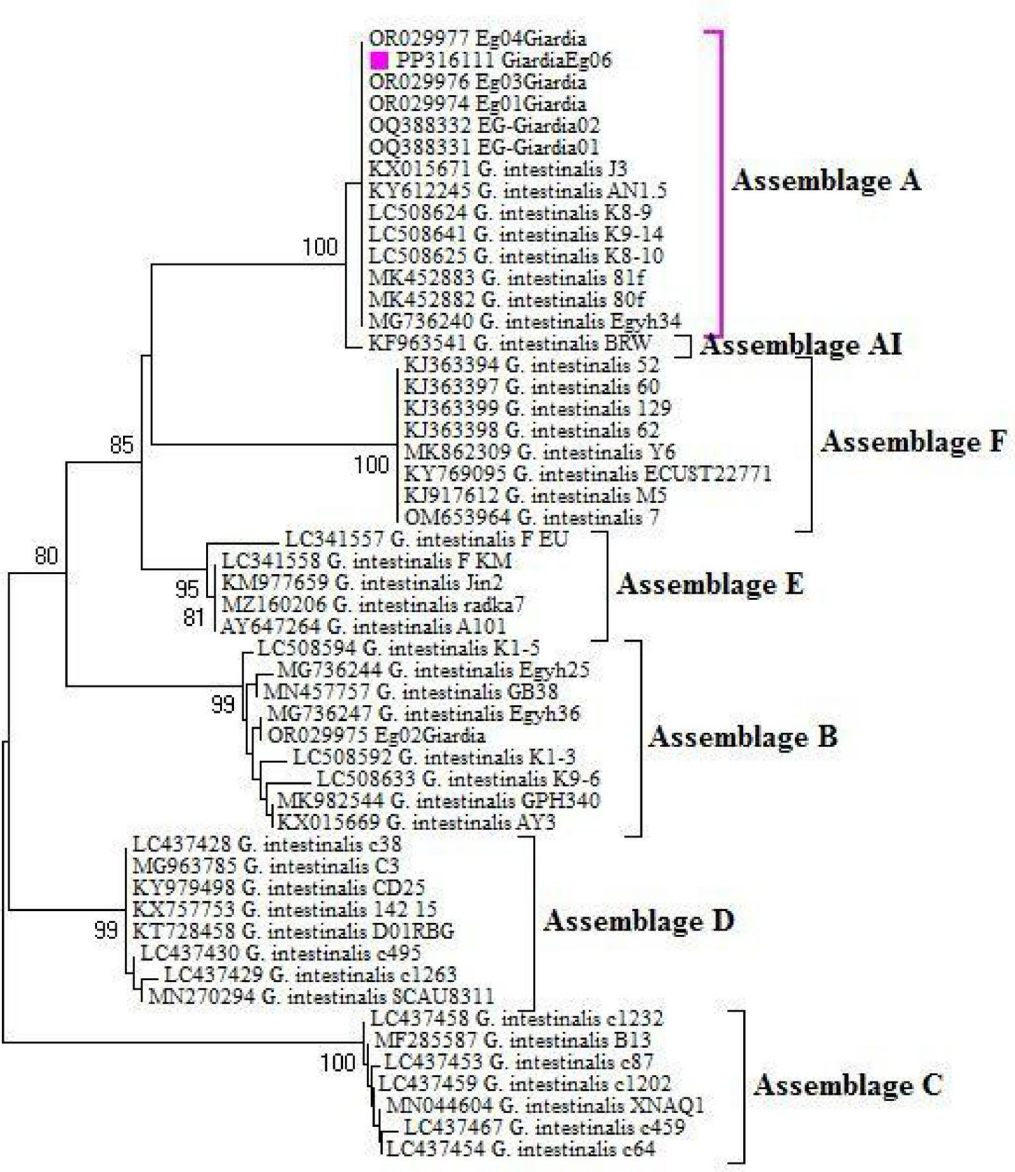
β-giardin -based phylogenetic tree of *Giardia* spp. The Maximum Likelihood method was constructed based on the Tamura-Nei model with 1000 bootstrap replicates.

All *Giardia*-positive samples were identified as *G. intestinalis* using *β-giardin* primers. BLAST analysis showed one genotype of *G. intestinalis* (GenBank: PP316111.1), with 100% identity (474/474 bp) to *G. intestinalis* detected in human feces from Brazil (GenBank: KX015671). However, the irrigation water sample yielded low-quality sequences, making identification challenging. Based on the β-giardin gene, the phylogenetic tree showed that our sequence clustered with other *G. intestinalis* of the assemblage A clade (Fig. 3).

## Discussion

Understanding the distribution and risk factors of *Cryptosporidium* and *Giardia* is crucial due to their significant role in zoonotic transmission and environmental contamination. Cattle, particularly pre-weaned calves, are major reservoirs, shedding large quantities of (oo)cysts, which contribute to environmental contamination and pose serious public health and veterinary risks^22,64^. Accurate species identification at the herd level is essential for implementing effective treatment and prevention strategies^65^. Therefore, this study aimed to investigate the prevalence of *Cryptosporidium* and *Giardia* infections, as well as co-infections, in cattle feces and nearby irrigation water in Beni-Suef Governorate, Egypt, using an integrated diagnostic approach combining microscopy and molecular techniques.

In this study, microscopic analysis revealed an overall infection rate of 67.42% for bovine cryptosporidiosis and giardiasis, with *Cryptosporidium* oocysts detection in 42.68%, *Giardia* cysts in 11.96%, and co-infections in 12.78% of the examined cattle. The prevalence of cryptosporidiosis was comparable to the 46% infection rate reported in cattle from Cairo, Giza, and Beni-Suef^66^ but was relatively higher than rates recorded in other Egyptian Governorates, such as 34.33% in Minufiya^67^38.27% in Upper Egypt^49^ and 24.67% in Kafr ElSheikh, 14.29% in Qalyubia, and 17.14% in Gharbia^68^. Globally, cryptosporidiosis prevalence varies widely, with reported rates of 1.61% in China^69^ 4.4% in Korea^70^, 8.3% in Kenya^71^ 10% in Ethiopia^72^ 13.7% in Algeria^73^ 19.23% in Saudi Arabia^48^ 27.3% in Canda^74^ and 55.4% in Austria^75^. These variations may be influenced by hygienic practices, infection severity, geographic location, cattle breed, animal age, seasonal factors, and sample size^76^.

Similarly, the giardiasis infection rate (11.96%) observed in this study is consistent with a previous report from El-Dakahlia, El-Gharbia, and Damietta Governorates, where a prevalence of 13.3% was recorded^77^. Globally, giardiasis prevalence in calves varies considerably, with reported rates of 2.1% and 2.2% in China^69,78^ 5.6% and 12.7% in Korea^70,79^ 5.7% in Bangladesh^80^ 7.5% in Brazil^81^ 27.1% in Austria^75^ 27.5% in Algeria^73^ 33.5% in the USA^82^ 39% in Ethiopia^72^ and 42% in Canada^74^. These variations in prevalence may be attributed to differences in geographic location, climate, herd management practices, diagnostic methods, and sample size^27^.

Co-infection with *Cryptosporidium* and *Giardia* was detected in 12.78% of studied cattle, consistent with previous reports indicating that such co-infections are common in bovines^83^. A higher prevalence (36%) was recorded in Ismailia Governorate, Egypt^84^ while lower rates were reported in Canada 8.5%^74^ and Austria 11.8%^75^. Moreover, several studies suggested a positive association between *Cryptosporidium* and *Giardia* infections, which may be linked to water contamination as a shared transmission route^85–87^.

Water was identified as a key risk factor for animal exposure to *Cryptosporidium* and *Giardia* in this study. *Cryptosporidium* spp. was detected in 2 of 24 irrigation water samples (8.33%), while *Giardia* was identified in 1 sample (4.16%). In Egypt, previous studies have reported *Cryptosporidium* and *Giardia* prevalence in water ranging from 5.2 to 80% and 13.6–100%, respectively^83,86–91^ with the highest prevalence observed in raw wastewater and the lowest in treated water^83,90^. Detecting these protozoan parasites in water remains challenging due to the complexity of the water matrix, the typically low concentration of (oo)cysts^92^ variations in contamination levels, and differences in water sources used for irrigation^93^.

The analysis of epidemiological risk factors revealed a significant association between animal sex and the prevalence of both overall infection and cryptosporidiosis, with female calves exhibiting higher infection rates. These findings are consistent with previous studies that have also reported a greater prevalence of cryptosporidiosis among female animals^94–96^. This increased susceptibility may be attributed to physiological and hormonal differences, as well as management practices such as the preferential retention of female calves for breeding, which may result in prolonged housing, higher stocking densities, and increased pathogen exposure.

In addition to sex, age also plays a critical role in infection susceptibility. Calves younger than 2 months showed higher morbidity rates for both overall infection and cryptosporidiosis, a trend that aligns with previous studies reporting increased susceptibility in this age group^77,94,95,97^. This heightened vulnerability is likely due to an underdeveloped and immature immune system^96^. Conversely, calves between 4 and 6 months of age exhibited a higher prevalence of giardiasis, which is consistent with findings indicating that giardiasis is most common in calves aged 2 months and older^75,77,98^.

Beyond age and sex, seasonal variations also influenced infection patterns. While both overall infection and giardiasis were more prevalent in the spring, cryptosporidiosis cases peaked in the autumn. These findings are somewhat variable across studies, as some have reported a higher prevalence of infection during the rainy season^99^ whereas others have noted increased cases in the summer^100^. These divergences may be attributed to regional differences in farming practices, environmental conditions, and the availability of resources to minimize contamination^30,96^.

Furthermore, clinical signs such as diarrhea were strongly associated with cryptosporidiosis, with diarrheic cattle exhibiting a higher prevalence of infection compared to non-diarrheic ones. This observation aligns with previous studies that have established diarrhea as a predominant clinical sign of cryptosporidiosis^94,99,101,102^.

Molecular analysis confirmed the presence of *Cryptosporidium* and *Giardia* species in cattle feces and irrigation water, underscoring the complex transmission dynamics of these protozoan parasites within the One Health framework^22,103,104^. Four *Cryptosporidium* species were identified, *C. hominis* and *C. bovis* in cattle feces, and *C. ubiquitum* and *C. ryanae* in irrigation water, highlighting the diversity of species circulating in animal and environmental reservoirs. Notably, this study represents the first detection of *C. hominis* in cattle in Egypt, a significant finding given that *C. hominis* is primarily associated with human infections^105^. Its presence in cattle suggests potential anthroponotic transmission, likely resulting from environmental contamination or direct human-cattle interactions, consistent with previous reports of cross-species transmission^20^. In Egypt, *C. hominis* has been documented in humans^106,107^ and recently in sheep^66^. Globally, *C. hominis* has been detected in various animal hosts, including cattle, sheep, goats, horses, donkeys, and camels^105^. The detection of *C. bovis* in cattle feces from Beni-Suef Governorate further supports the role of livestock as reservoirs, contributing to environmental contamination and potential zoonotic transmission. Previous studies in Egypt have reported *C. bovis* in cattle from different Governorates, including Ismailia^108^ Kafr El Sheikh^33,109^ Beheira, Menofia, Qaliubiya, Assiut, and Sohag^110^. Consistent with global trends, *C. bovis* is commonly detected in cattle populations, often with low or no occurrence of *C. parvum*, as observed in Sweden^111^ China ^112^ Australia^113^ and Canada^114^.

The identification of *C. ubiquitum* and *C. ryanae* in irrigation water underscores the significance of waterborne transmission pathways. To the best of our knowledge, this study represents the first molecular detection of both species in irrigation water in Egypt. Previously, only *C. parvum and C. hominis* have been reported in drinking water in the country^86,90,115^. *Cryptosporidium ubiquitum* is recognized as the most prevalent *Cryptosporidium* spp. in sheep and goats^20,116–118^ and is also emerging as a human pathogen^119^. In Egypt, *C. ubiquitum* has previously been detected in sheep^120^. Similarly, *C. ryanae* is primarily associated with cattle^20,121^ and has been reported in cattle^33,77,108,109^ and buffaloes^108,109,122^ in Egypt. The detection of these species in irrigation water suggests that runoff from livestock operations may be a significant source of environmental contamination. This finding highlights the urgent need for water quality monitoring and improved agricultural waste management strategies to mitigate the risk of protozoan transmission through irrigation systems.

In this study, the identification of assemblage A, further supports the risk of cross-species transmission. In Egypt, *Giardia* assemblage A has been previously reported in cattle^77^ humans^42,123–125^ and tape water in the Beni-Suef Governorate^86^. The inability to obtain high-quality sequences from irrigation water suggests that environmental factors, such as microbial competition or DNA degradation, may influence *Giardia* detectability in water sources^93^. Nevertheless, the presence of *G. intestinalis* in cattle feces, along with its previous detection in tap water from the same region^86^ indicates that livestock may serve as reservoirs, with potential transmission occurring through direct contact, fecal contamination of water sources, or consumption of contaminated agricultural products.

## Conclusion

This study highlights the high prevalence of *Cryptosporidium* and *Giardia* infections in cattle and irrigation water in Beni-Suef Governorate, Egypt, underscoring the role of cattle as reservoirs and the risk of environmental contamination. The first detection of *C. hominis* in cattle and *C. ubiquitum* and *C. ryanae* in irrigation water in Egypt suggests potential anthroponotic and waterborne transmission pathways while confirming the presence of *C. bovis* and *Giardia* assemblage A in cattle. Risk factor analysis showed higher infection rates in females, young calves, and during spring, with diarrheic feces strongly linked to parasite shedding. These findings emphasize the need for enhanced surveillance, improved livestock management, and stricter water quality monitoring. This study has some limitations, including its cross-sectional design, limited PCR sensitivity due to low (oo)cyst counts, and the lack of direct evidence linking water contamination to animal infection. Further research is needed to clarify transmission pathways and assess long-term impacts. Implementing *One Health* strategies with targeted interventions is essential to reducing infection risks and environmental contamination.

## Electronic supplementary material

Below is the link to the electronic supplementary material.

Supplementary Material 1

## Data Availability

Data availability: All data generated and analyzed in this study are included in the published manuscript. The nucleotide sequences of *C. hominis, C. bovis, C. ubiquitum,* and *C. ryanae* for the *18S* rRNA gene and *G. intestinalis* for the *β-giardin* gene obtained in this study have been submitted to the GenBank, GenBank accession numbers: PQ149132.1, PQ149134.1, PQ149133.1, PQ149135.1 and PP316111.1 (https://www.ncbi.nlm.nih.gov/genbank).
