# Supplementary material for: Coprological and molecular prevalence of Cryptosporidium and Giardia in cattle and irrigation water from Beni-Suef Governorate, Egypt
Source: Sci Rep. 2025 Jul 24;15:26983. doi: 10.1038/s41598-025-10552-7 (PMC12289956; doi:10.1038/s41598-025-10552-7)
Supplement: Supplementary file 1 — Supplementary Material 1 [file 41598_2025_10552_MOESM1_ESM.docx]

**Supplement Material**


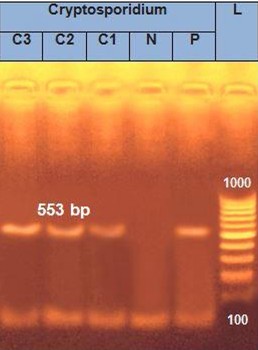


**S1 Appendix Fig.1:** Electrophoresis gel of the 18S rRNA gene amplification for *Cryptosporidium* spp. L: 100 bp DNA ladder; P: positive control; N: negative control; C1–C3: positive *Cryptosporidium* spp. samples. The expected amplicon size is 553 bp.


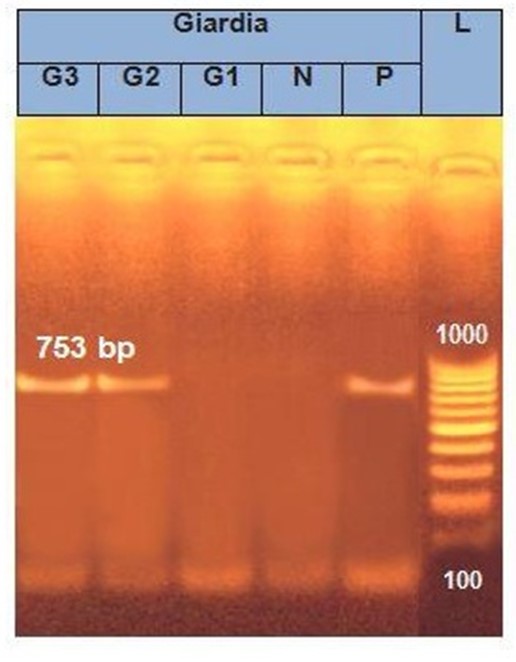


**S2 Appendix Fig.2:** Electrophoresis gel of the β-giardin gene amplification for *Giardia* spp. L: 100 bp DNA ladder; P: positive control; N: negative control; G1–G3: positive *Giardia* spp. samples. The expected band size is 753 bp.
